# What Is the “Trigger” of Addiction?

**DOI:** 10.3389/fnbeh.2020.00054

**Published:** 2020-04-21

**Authors:** Samuel Asensio, Vicente Hernández-Rabaza, José Víctor Orón Semper

**Affiliations:** ^1^Departamento Ciencias Biomédicas, Universidad Cardenal Herrera-CEU Universities, Valencia, Spain; ^2^Instituto de Ciencias Biomédicas, Universidad Cardenal Herrera-CEU Universities, Valencia, Spain; ^3^Centro Universitario Santo Tomas, Universidad Católica de Ávila, Ávila, Spain

**Keywords:** trigger, addiction, subjective, frustration, treatment

Addiction is a multidimensional condition (European Monitoring Centre for Drugs Drug Addiction., 2014) which has traditionally been explored from different perspectives, including biological, social, and psychological approaches. Inside of these main levels, the specific contribution of multiple sublevels to the addiction development and treatment have been investigated. This research field draws an amazing addiction puzzle which is built by a diverse set of pieces coming from the genetic, epigenetic, molecular, neurobiological, and psychological levels, but also from live experiences, the environment, and from cognitive traits. The complex combination of these factors determines the addiction process.as well as Drug use affects each factor differently.

Any attempt to study this complexity through one single level is insufficient. In fact, addiction models usually need to include elements from different models in order to provide a satisfactory explanation of the disease.

Most addiction research has classically focused on the neurobiological level, trying to figure out the neuroadaptations that repetitive drug use produces over the brain systems and their behavioral consequences, including effects on the reward system, emotional or cognitive functioning. Currently, one of the most accepted neurobiological theories postulates that the development of drug addiction is a progressive process through a three-phase cycle: binge/intoxication, withdrawal/negative affect and preoccupation/anticipation (Koob and Volkow, 2016).

However, there is no doubt that addictive disorders have a strong subjective component that is not fully fitted with the present models. There is increasing literature showing how some factors related to subjective processes can impact the neurobiology of addiction by increasing the vulnerability such as early childhood experiences (Kim et al., 2017; Lee et al., 2018), social context (Schriber and Guyer, 2016; Burke et al., 2017), environment (Zucker et al., 2018), maturation (Romer et al., 2017) or personality (Jauk and Dieterich, 2019; Ramirez-Castillo et al., 2019) factors. Of special interest is the role of the so called “Big Five” personality traits in the risk for drug addiction (Andreassen et al., 2013).

## Re-Understanding the Trigger

The current maladjustment between the neurobiology and the subjective human condition can be observed in the concept of “trigger.” Understood as “a stimulus that elicits a reaction” (APA dictionary, 2019), the trigger is considered a key element in the craving response showed by addicts. This external stimulus would lead the individual to repeat drug use or relapse after a period of abstinence. Addiction models constructed upon this observation consider the trigger as a stimulus able to activate drug related memories leading to reward anticipation and craving responses. As a consequence, derived therapeutic approaches suggest to avoid the trigger or provide the individuals with cognitive capabilities to control that emotional response provoked by the trigger. Such cognitive-behavioral therapies include operant conditioning, contingency management or coping skills training (Witkiewitz et al., 2019).

In this way, where “trigger” is considered as an “external” stimulus inducing a reaction, its scope is only at the psychological level and does not address the uniqueness of complexity. Instead, we offer a re-understanding of the “trigger” as something “internal” that relates all levels of complexity and requires dialogue between different levels mentioned above. Moreover, the stimulus-response association was already questioned by PK Anokhin (Egiazaryan and Sudakov, 2007), who proposed to come out of a causal reading (the trigger provokes a behavior) and assumed a systemic conception in which the behavior is due to a global situation of the whole system (Thelen and Smith, 1994; Smith, 2005; Anderson et al., 2012).

The change from outside to inside is also justified by discovering that due to the high subjectivity of addiction, it makes no sense to “blame” something outside. In this sense, a term to refer to all this subjective complexity is suggested here: “frustration.” Frustration cannot be understood without breaking expectations (Amsel, 1992). The interesting point about this term is that it evidences aspects of interiority, but its conceptual basis also allows its use in the different levels of complexity. If we define frustration as widely as possible, we would say that it is the emotional result of the perception of the distance between the expected (needed) and the found. At all levels, even in the most molecular, frustration would indicate a mismatch between one molecular situation and another. Frustration is understood as a global experience that can have many types of “mismatches,” whether molecular or in terms of expectations. This mismatch needs to be perceived either by cognitive (if we talk about expectations) or biological processes (if we talk about biological levels). Frustration would thus be a meeting point for all levels of complexity.

Classically, frustration and other emotions were considered as an evaluation of the actual need and estimation of probability of its satisfaction (the “need-informational theory of emotions”), linked to the participation of specific key brain structures (Simonov, 1984, 1997). However, recent meta-analyses found little evidence that discrete emotion categories can be consistently and specifically localized to distinct brain regions (Lindquist et al., 2012). Therefore, a set of interacting brain regions commonly involved in basic psychological operations are active during emotion experience and perception across a range of discrete emotion categories (Kober et al., 2008; Lindquist et al., 2012). Therefore, the current model of emotions is systemic and linked to other psychological functions (Pessoa, 2013), which is in line with the proposal of “frustration” as a personal global condition which (after the whole personal evaluation of a stimulus) “triggers” a response.

What is usually conceptualized as a trigger (cause attributed to an external element) would be a simplification because it denies the role of the inner experience. If we accept the frustration is previous to the external trigger, then the clinical approach should be headed to search and treat the emotional “tangle” underlying frustration and its relationship with the external stimulus. Because if we avoid the external trigger without treating the previous subjective cause, then the probability of relapse is high. The treatment of the subjective emotional state will help to provide a new meaning to that external stimulus, an action that we call to “re-meaning” the trigger.

This therapeutic fact of giving a new meaning to the trigger does not exclude the traditional therapeutic avoiding of the trigger, which is an urgent aim at the beginning of the treatment. Nevertheless, after that initial phase, the inner problem should also be addressed. Actually, both are necessary, one to get initial abstinence and the other to help the addict to resolve the frustration underlying drug addiction.

**Figure 1:**
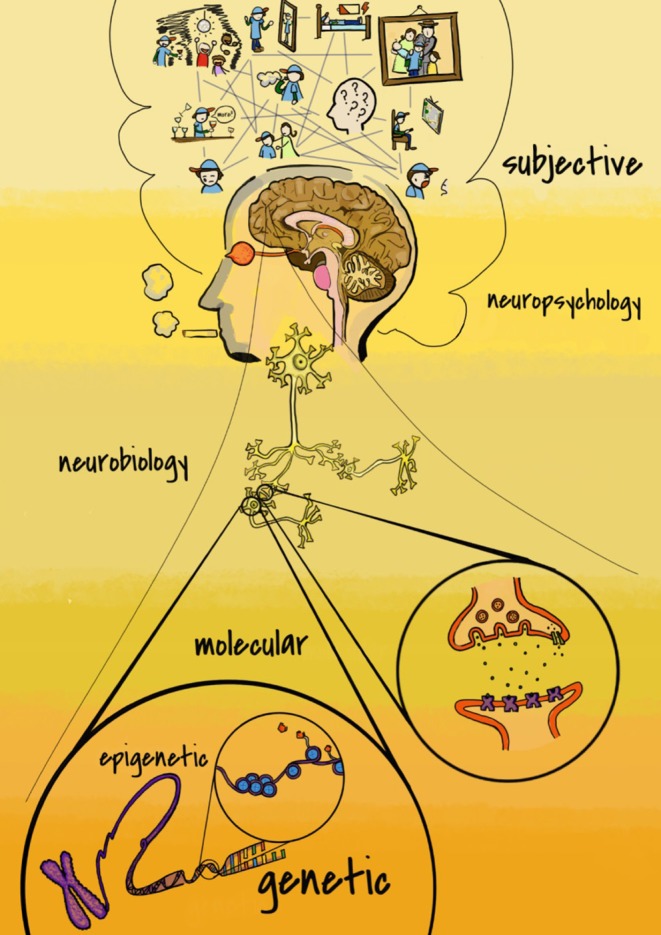
Different levels for the study of addiction range from molecular **(lower)** to subjective **(upper)**.

Our proposal about the individual subjectivity implies understanding that the emotion is lived more as an expression of the complexity of a person's life in specific circumstances, than as a reaction to the outside (Barrett, 2017).

## The Subjective Concept and Neurobiology of Stress and Addiction

Similarly to addiction, stress disorders are also related to a trigger stimulus evoking a strong subjective experience. A neurobiological overlap between these two conditions would therefore be expected.

The responses to psychosocial stressful stimuli in healthy individuals also involve the participation of hippocampus, amygdala, insula and prefrontal cortices (Shin and Liberzon, 2010). Specifically, limbic circuits underlie the coherent contextualization of different neural inputs (Bird, 2017), as well as the formation of episodic memories and the integration of emotional processing; essential elements in craving and relapse by exposure to the context of drug addicts.

Moreover, drug addicts, psychiatric patients, and subjects that suffered early child abuse show similar brain alterations such as volume reductions of the hippocampus, amygdala and anterior cingulate cortex, or hyperactivity of the amygdala and insula, vs. a decreased response of prefrontal cortex when dealing with stress (Etkin and Wager, 2007; Shin and Liberzon, 2010; McCrory et al., 2012). The cue-reactivity paradigm used in fMRI addiction studies has pointed out limbic and prefrontal cortices as the key systems in response to stimuli (Chase et al., 2011). However, a more recent meta-analysis concludes the absence of a consensus in relation to the brain response to conditioned drug stimuli (Zilberman et al., 2019). The loss of consensus can possibly be partially explained by the role of frustration (subjective personal factors) triggering the negative perception of the reality (inner trigger), an element shared in both, stress and addiction disorders.

Our suggestion is to promote resilience as a therapeutic tool to treat frustration. It is known that the subjective perception of the event is a determining point to understand the experience lived (Burr, 1982). Therefore, the best way to work on resilience is through the re-meaning of the so-called stressor or trigger (Lazarus and Launier, 1978; Boss, 2002), but working on the meaning attributed to the stressor instead of the stressor itself. A creative act is necessary because resilience is not a mere adaptation to new circumstances, but implies a global personal growth (Walsh, 2002; Cicchetti, 2010).

From a therapeutic view, the capability of psychotherapeutic treatments (alone) has been demonstrated to restoring the biological normality of brain structure and function (Barsaglini et al., 2014). This is of especial interest when only limited effects have been documented by pharmacological treatments, for example in the drug addiction (Dakwar and Nunes, 2016). Psychological symptoms, including depression, anxiety, hostility, psychological pain, embarrassment, blame, panic and obsession, are complex and difficult to characterize but treating them is crucial and essential for rehabilitation (Dakwar and Nunes, 2016).

## Turning Back to the Complexity

Usually, the emphasis for relapse prevention is focused on avoiding trigger stimuli by means of healthy habits, but, once again, subjective elements play a central role, and are related to the complexity of personal relationships and self-assessment (Marlatt and Gordon, 1985). Therefore, relapse is seen as the effect of not having coping strategies. Moreover, it has been shown that high percentages of drug addicted patients allege intrapersonal determinants related to frustration as the main cause of relapse (Ramirez-Castillo et al., 2019).

It is clear that resilience to stress or addiction must be studied at all levels from the most biological to the most subjective (Cicchetti, 2010) in order to attend the globality and uniqueness of the person since the absence of risk factors or the presence of protective elements alone are not enough to explain whether an individual using drugs will become addicted or whether an addict will be rehabilitated (Luthar et al., 2000).

This brief journey opens the possibility of accepting the term “frustration” as a global subjective element, leading the therapeutic intervention toward the inner patient condition, for example, through work on the resilience, more than the avoiding of external stimuli.

## Author Contributions

All authors listed have made a substantial, direct and intellectual contribution to the work, and approved it for publication.

## Conflict of Interest

The authors declare that the research was conducted in the absence of any commercial or financial relationships that could be construed as a potential conflict of interest.
